# Research on improved level set image segmentation method

**DOI:** 10.1371/journal.pone.0282909

**Published:** 2023-06-21

**Authors:** Mei Zhang, Dan Meng, Lingling Liu, Jinghua Wen

**Affiliations:** 1 Information Institute, Guizhou University of Financial and Economics, Guiyang, China; 2 Guizhou Key Laboratory of Big Data Statistical Analysis (No.[2019]5103), Guiyang, China; University of Engineering & Technology, Taxila, PAKISTAN

## Abstract

Aiming at the shortcomings of the traditional level set model which only has good robustness to the weak boundary and strong noise of the original target image, this paper proposes an improved algorithm based on the no-weight initialization level set model, introducing bilateral filters and using implicit surface level sets to extract and segment the original target image object more accurately, clearly and intuitively in the evolution process. The experimental simulation results show that, compared with the traditional non-reinitialized level set model segmentation method, the improved method can more accurately extract the edge contours of the target image object, and has better edge contour extraction effect, and the original target noise reduction effect of the improved model is better than that of the model before the improvement. The original target image object edge contour takes less time to extract than the conventional non-reinitialized level set model before the improvement.

## 1. Introduction

Image segmentation is a fundamental work with important research value within the field of image processing and computer vision research [1]. Up to now, there is still no universal image segmentation method that can cope with or solve the segmentation problem of all original target images. Many studies and improvements on image segmentation algorithms exist at home and abroad, especially in the field of computer vision and information processing research [2].

Based on the existing classical image segmentation definitions, image segmentation can be roughly classified into region-based image segmentation algorithms (region growth method [3], clustering method [4] and boundary-based image segmentation algorithms [5] using intra-region similarity (e.g. Robert operator, Sobel operator, Laplacian operator, Canny operator). Deep learning methods have been mainstream methods of image processing:Wei Tang et al. propose a novel infrared and visible image fusion method, i.e., the Y-shape dynamic Trans- former (YDTR). Specifically, a dynamic Transformer module (DTRM) is designed to acquire not only the local features but also the significant context information [6]; Wei Tang et al. propose a novel unsupervised method to fuse multimodal medical images via a multiscale adaptive Transformer termed MATR. Instead of directly employing vanilla convolution, they introduce an adaptive convolution for adaptively modulating the convolutional kernel based on the global complementary context [7].

There is no shortage of optimization and improvement of various level set related series of image segmentation algorithms.

Bembenek Michal use a multiclass level-set approach with a developed penalty term as a framework for the advanced final damage segmentation stage, a quantitative analysis of the accuracy of the proposed approach is provided, the efficiency of the approach is demonstrated on authentic images of coated surfaces [8]. Birane Abdelkader et al. have developed a new level set function to implement a fast and robust active contour model. The model was built by combining the sbgfrls model and the Legendre polynomial, and instead of using the average intensity of the region, the level set function was regularized using a Gaussian filtering process [9]. Shamsi Koshki Asma et al. proposed a new local region based active contour model, the local self-weighted active contour model. The experimental results of applying the model to include synthetic and medical images, especially breast thermography images, were compared with the well-known local level set approach and demonstrated the perfect ability of the model [10]. Weng Guirong et al. proposed an additive bias correction (ABC) model based on intensity non-uniformity, which converts the local clustering criterion into an energy function based on the level set model by introducing a level set function [11].

Arora Jyoti et al. design an integrated method to implement a level set segmentation process for medical image segmentation that helps overcome the problem of manually initializing parameters. The input image is simplified by an intuitive fuzzification process of the image. Further segmentation is performed using an intuitive clustering technique with local spatial information (S-IFCM), and the control parameters of the level set method are automatically implemented by S-IFCM [12]. Badshah Noor et al. propose a first-hand mixing model via a mixing kernel and a Euclidean distance metric. Experimental results on a number of real and synthetic images show that the proposed model outperforms the models of Chan and Vese [13]. Chen H et al. proposed a fast image segmentation algorithm with a parametric level set active contour model, which embeds the parametric level set function into the classical LGDF (local Gaussian distribution fitting) model for image segmentation without the need for re-initialization and additional regular terms [14].

Hsieh Chi-Wen et al. developed an effective and accurate active contour model by improving the Distance Regularized Level Set Evolution (DRLSE) model to improve segmentation ability. The concept of balancing the thrust and image contour strength of the DRLSE model for contour segmentation was used to redefine the contour convergence / divergence forces to make it more feasible for complex boundaries [15]. Mesadi Fitsum et al. proposed a new parametric level set method, the Disjunctive Normal Level Set (DNLS), and applied it to two-phase (single-object) and multiphase (multi-object) image segmentation formulated segmentation algorithms in a Bayesian framework and used variational methods to minimize the energy relative to the model parameters [16]. Jiang Xiaoliang proposed a level set image segmentation algorithm with a local cross-entropy measure of fuzzy C-means and its simplified model. Compared with the traditional level set algorithm, this method successfully handles weak edges and grey scale inhomogeneous targets with some noise immunity [17].

On the whole, there are few algorithms for optimizing the weak boundary and strong noise robustness range of traditional uninitialized level sets. In this paper, we analyze and study the advantages of bilateral filters in the process of image noise reduction, improve the noise reduction method of the level set model in the pre-processing stage of the original target image, and further optimize and improve the existing original target image segmentation model in view of the defects of the level set model in the aforementioned research status. The scope of the no-weight initialization level set model, which is only robust to the weak boundary and strong noise of the original target image, is extended, so that the scope of weak boundary extraction and strong noise reduction of the original target image is expanded to a certain extent. In this thesis, the original target image is improved and optimized to some extent under the no re-initialization level set algorithm, and finally simulation experimental programming is carried out to verify the effectiveness of the improved algorithm.

## 2. Level set model without re-initialization

Osher et al. proposed the Level Set Method (LSM) based on the theory of the evolution of the edge profile curve of the original target object [18]. The basic idea of the Level Set Method is to complete the process of evolving the edge profile curves of the original target image objects by using the continuous updating of the high-dimensional implicit function. The evolution curve of the object edge profile of the original target image is assumed to be *C*(s, *t*), where s the parameter variables used to represent the object edge profile curve of the original target image are represented, and the time variables t used to represent the evolution curve are represented; the implicit level set function of the higher dimension is assumed to be *ϕ*(*x*, *y*, *t*); if *ϕ*(*x*, *y*, *t*) = *ϕ*(*C*(*s*, *t*), *t*) = 0 and when, that is, the zero level set of the evolution curve of the object edge profile of the original target image *C*(s, *t*) at the moment t. In general, there are different forms of level set function descriptions, but in order to consider the numerical stability of the object edge profile evolution curve of the original target image in the solution process, the Signal Distance Function (SDF) is usually used as the main form of description. The definition of the SDF is shown in Eq (1) [19]:

$$\phi (x,y,t)=\left\{\begin{array}{c} -d,(x,y)inside\;C(s,t)\\ 0,(x,y)at\;C(s,t)\\ d,(x,y)outside\;C(s,t) \end{array}\right.$$ (1)

From Eq (1), it can be derived that d is *ϕ*(*x*, *y*, *t*) the distance from the edge profile evolution curve of the original target image object *C*(*s*, *t*) to its embedded higher one-dimensional implicit level set function; when *ϕ*(*x*, *y*, *t*) the point of the implicit level set function (*x*, *y*) lies inside the edge profile evolution curve *C*(*s*, *t*) of the original target image object, then the opposite *d* of the distance is taken as the value *ϕ*(*x*, *y*, *t*) of the implicit level set function; when the point (*x*, *y*) of the implicit level set function *ϕ*(*x*, *y*, *t*) is located on the original target image object edge contour evolution curve *C*(*s*, *t*), then the original target image object edge contour evolution curve *C*(*s*, *t*) is the zero level set of the level set function *ϕ*(*x*, *y*, *t*), that is, the distance *d* is taken to be 0 as the value of the implicit level set function *ϕ*(*x*, *y*, *t*) at this time; when the point (*x*, *y*) of the implicit level set function *ϕ*(*x*, *y*, *t*) is located outside of the original target image object edge contour evolution curve *C*(*s*, *t*), the value of the distance *d* is taken as the value of this implicit level set function *ϕ*(*x*, *y*, *t*) at this time.

Using the principle of full differentiation to derive the parameters *t* of the time variables *ϕ*(*x*, *y*, *t*) = *ϕ*(*C*(*s*, *t*), *t*) = 0 in the level set function, Eq (2) is obtained [20]:

$$\frac{\partial \phi }{\partial \text{t}}+\nabla \phi \cdot \frac{\partial C}{\partial t}=0$$ (2)

In Eq (2), the evolution equation of the edge profile curve *C*(*s*, *t*) of the target object of the original image is defined as Eq (3):

$$\frac{\partial C}{\partial t}=V\vec{N}$$ (3)

In Eq (3), *V* is a representation of the evolution rate of the edge profile curve *C*(*s*, *t*) of this original target image object, and $\vec{N}(\vec{N}=-\nabla \phi /|\nabla \phi |)$ is a representation of the inward unit normal vector of the edge profile curve *C*(*s*, *t*) of the original target image object. By substituting the evolution equation and $\vec{N}$ the value of the inward unit normal vector for the evolution equation *C*(*s*, *t*) of the edge contour curve of the given original target image object into the level set full differential equation derived for the time variable parameter *t*, the evolution equation of object edge contour curve of original target image based on level set can be finally transformed into Eq (4), as shown below:

$$\frac{\partial \phi }{\partial \text{t}}-V|\nabla \phi |=0$$ (4)

## 3. Improved level set image segmentation

### 3.1 Improving algorithmic foundations

The curve *ϕ*((*C*(*t*), *t*)): *R*^2^ × *R*^+^ → *R* is assumed to be a level set function, which is an implicit expression of a *t* moment-to-moment continuous closed curve *C*(*t*), usually taken *ϕ*((*C*(*t*), *t*)) = 0 as the level set of the zero equivalence line. Derivation of the function *ϕ*((*C*(*t*), *t*)) = 0 according to the principle of full differentiation yields the formula (5) [17]:

$$\frac{\partial \phi }{\partial \text{t}}+\nabla \phi \frac{\partial C}{\partial t}=0$$ (5)

Let s be the arc length parameter *C* of the edge contour curve of the original target image object, the amount of variation of the implicit level set function *ϕ* along the tangent direction of the edge contour curve *C* of the original target image object satisfies the following relation, see Eq (6):

$$0=\frac{\partial \phi }{\partial x}\frac{\partial x}{\partial s}+\frac{\partial \phi }{\partial y}\frac{\partial y}{\partial s}=<\nabla \phi ,\frac{\partial C}{\partial s}>$$ (6)

If the implicit level set function *ϕ* takes negative values inside the original target image object edge contour curve *C* and positive values outside it, the basic equations for its internal unit normal vector and level set evolution method are expressed as Eqs (7) and (8), respectively:

$$\vec{N}=-\frac{\nabla \phi }{\left|\nabla \phi \right|}$$ (7)

$$\frac{\partial \phi }{\partial t}=F|\nabla \phi |$$ (8)

Eq (6) belongs to the Hamilton-Jacobi equation. Taking the negative sign of the inner unit normal vector $\vec{N}$ in Eq (4), it can be derived that: when the level set implicit function *ϕ*(*x*, *y*, *t*) > *c*, (*x*, *y*), (*x*, *y*) is located outside the original target image object closed edge contour curve *C*; when the level set implicit function *ϕ*(*x*, *y*, *t*) < *c*, (*x*, *y*), (*x*, *y*) is located inside the original target image object closed edge contour curve *C*; when the level set implicit function *ϕ*(*x*, *y*, *t*) = *c*, (*x*, *y*), (*x*, *y*) is located in the original target image object closed edge contour curve *C*.

Usually the *c* = 0 time is taken to represent the embedding of the evolution curve of the edge contour of the object of the selected original target image by an implicit function zero level set of one dimension higher [21]. In the segmentation process of the original target image, the description of the initial evolution curve of the edge contour of the selected original target image object is usually represented using the zero level set function defined in the original target image region, and the continuous updating of the zero level set function is accomplished by the combination of Eq (8), which drives the evolution curve *C* of the edge activity contour of the original target image object toward the selected original target image object edge contours continuously.

Caselles and Malladi were the first to propose curve evolution theory and the level set approach model separately. Since the level set approach model remains stable during the evolution of the selected original target image object edge contour curves, periodic initialization of the level set function *ϕ*(*x*, *y*) is required [22], which leads to a significant increase in computational effort during the evolution of the original target image object edge contour curves. Assuming Ω that the entire original target image domain is represented and the image intensity is *I*(*x*, *y*): Ω →*R*, the closed selected object edge contour curve *C* in the original target image Ω is represented implicitly by the high one-dimensional level set function *ϕ*(*x*, *y*, *t*) zero level set, i.e. *C* = {(*x*, *y*)|*ϕ*(*x*, *y*, *t*) = 0}. The initial representation of the signed distance function generated by the curve *C* is shown in Eq (9) (generally taken as positive for the inner side of the curve and negative for the outer side) [23]:

$$\phi (x,y,0)=\left\{\begin{array}{c} d,\text{if}\;x\;is\;inside\;C\\ 0,if\;x\;is\;on\;C\\ -d,if\;x\;is\;outside\;C \end{array}\right.$$ (9)

In Eq (9), *d* is the distance from the point (*x*, *y*) in the plane image to the *ϕ*(*x*, *y*, *t*) zero level set of the level set function.

The level set approach in the driven evolution of the partial differential equation can thus be obtained as a formula for the process of re-initializing the level set function, as shown in Eq (10) for the solution of the partial differential equation:

$$\frac{\partial \phi }{\partial t}=sign(\phi_{0})(1-|\nabla \phi |)$$ (10)

In Eq (10), which *ϕ*_0_ is the level set function that needs to be initialized again, *sign*(*ϕ*_0_) is the symbolic function, and *ϕ* is the level set function after the initialization is *ϕ*_0_ completed. Both the period of the re-initialized level set function and the choice of step size will affect the initialization of the level set function during the iteration to some extent [24].

This can be inferred from the nature of the symbolic distance function in the variational level set, and if the level set function |∇_*ϕ*_| ≈ 1, then the level set function *ϕ* meets the symbolic distance function requirement. It follows that the Li model defines the integral equation shown in Eq (11) as the penalty energy term (also known as the internal energy term):

$$E_{p}(\phi )=\iint_{\Omega }\frac{1}{2}{\left(|\nabla \phi |-1\right)}^{2}dxdy$$ (11)

Eq (11) is a measure of the proximity of the level set function to the symbolic distance function during the evolution of the edge profile of the selected original target object. The internal energy term is introduced to constrain the initialized level set function *ϕ* to ensure that the level set function *ϕ* is always a valid symbolic distance function during the subsequent evolution of the edge profile of the selected original target object, successfully reducing the computational complexity of initializing the level set function *ϕ* again during the evolution.

### 3.2 Bilateral filters improving the traditional level-set model

The evolution equation for the evolution curve *C* of the object edge contour of the original target image for the geometric active contour model is expressed in Eq (12) [20]:

$$\frac{\partial C}{\partial t}=g(V_{0}+ak)\vec{N}$$ (12)

In Eq (12), *g* is the condition used to determine whether the points on the evolution curve *C* of the edge contour of the original target image object need to continue to move during the motion; *V*_0_ is used to represent a constant coefficient that acts similarly to a balloon force (when *V*_0_ > 0, the original target image object edge contour evolution curve *C* expands outward; when *V*_0_ < 0, the original target image object edge contour evolution curve *C* contracts inward); *a* is a constant value used to adjust the curvature intensity; *k* is used to denote the curvature of the original target image object edge profile evolution curve *C*, which can both maintain the smoothness of the original target image object edge profile curve during evolution and enhance the resistance to noise interference during the evolution of the original target image object edge profile curve; and $\vec{N}$ represents the inward unit normal vector of the evolution curve *C* of the object edge profile of the original target image. Since both the constant velocity and curvature of the evolution curve of the edge contour of the original target image object do not have a direct correlation with the preprocessed target image, the existence of the edge indicator function, i.e. the judgment condition *g*, does not influence the evolution motion of the edge contour curve *C* of the original target image object through the information of the preprocessed target image.

There are two main methods for smoothing and noise reduction of the original target image in the image segmentation process, one is the use of Gaussian filters, which are obtained by assigning different weights to the Gaussian values within a certain range of pixel values at the current point and then weighting and averaging them, i.e., the Gaussian kernel function is a radial basis function most often used, which only takes into account the influence of the spatial domain weights of the neighbouring pixels at the current point of the evolution curve of the edge contour of the selected original target image; another approach is to use a bilateral filter, which takes into account not only the spatial domain weight of the current point in the evolution process, but also the weight of the pixel grey value domain of the neighbouring pixel points. For example, in the evolution process of the edge contours of the selected original target image, in addition to the more common spatial distance of the neighbouring pixels, there are also relevant factors such as grey-scale similarity values.

Based on the differences between the above two image smoothing filters, it can be seen that the Gaussian filter does not adequately take into account the edges of the original target image object, especially when the edges of the original target image object change dramatically in grey scale; whereas the non-linear bilateral filter can effectively optimize and improve the above defects. In order to obtain a more intuitive and accurate result of extracting the edge contour of the original target image object, we replace the Gaussian filter in the unweighted initialized level set with a bilateral filter to complete the improvement of the unweighted initialized level set model.

The total energy function of the Li model is defined in Eq (13) [25]:

$$E(\phi )=\mu P(\phi )+E_{g,\lambda ,\nu }(\phi )$$ (13)

In Eq (13), (*μ* > 0) is the constraint strength constant that controls the penalty energy term on the level set function *ϕ* and determines the degree of approximation to *ϕ* its symbolic distance function. *E*_*g*,*λ*,*v*_(*ϕ*) is the external energy term that controls the evolutionary motion of the edge profile curve of the original target image object and is defined in the form of Eq (14):

$$E_{g,\lambda ,\nu }(\phi )=\lambda L_{g}(\phi )+\nu A_{g}(\phi )$$ (14)

In Eq (14), *λ*(*λ* > 0) and *v* are constants, *L*_*g*_(*ϕ*) and *A*_*g*_(*ϕ*) are defined as defined as Eqs (15) and (16) respectively:

$$L_{g}(\phi )=\int_{\Omega }g\delta (\phi )|\nabla \phi |dxdy$$ (15)

$$A_{g}(\phi )=\int_{\Omega }gH(-\phi )dxdy$$ (16)

In Eq (15), *δ*(*ϕ*) is the Dirac function and *H*(−*ϕ*) is the Heaviside function in Eq (16), and *g* is the edge guide function.

The form of the edge guidance function *g* before and after the improvement of the Li model is given in Eqs (17) and (18) respectively:

$$g=\frac{1}{1+|\nabla G_{\sigma }*I{|}^{2}}$$ (17)

In Eq (17), *G*_*σ*_ is a Gaussian kernel function with standard deviation *σ*,”*” denotes the convolution, and *I* denotes the image intensity at that pixel point.


$$g=\frac{1}{1+\left|{\left.\frac{1}{W_{p}}\nabla (G_{\sigma_{s}}\cdot G_{\sigma_{r}})*I\right|}^{2}\right.}$$ (18)

In Eq (18), 1/*W*_*p*_ is the normalization factor,$G_{\sigma_{s}}$ is a spatial domain kernel function, *σ*_*s*_ is used to filter the pixel space neighborhood size;$G_{\sigma_{r}}$ is the range-domain kernel function, *σ*_*r*_ controls the extent to which the adjacent pixel weights are reduced due to intensity;”*”denotes the convolution, and *I* denotes the image intensity at that pixel point.

This paper proposes an improved model of the Li model, using a bilateral filter to replace the contribution of the Gaussian filter in the smoothing phase of the model image, a rather important step in the overall image segmentation process. In this process, the extraction of the edge contours of the selected original target image objects is mainly performed by constantly balancing the spatial domain weights and the weights of the pixel range weight domain.

## 4. Experimental simulation and analysis of results

### 4.1 Data source selection

In this paper, the accuracy and validity of the algorithm is verified by using experimental simulations for both the traditional unweighted initialized level set [25] and the improved unweighted initialized level set models.

**Fig 1:**
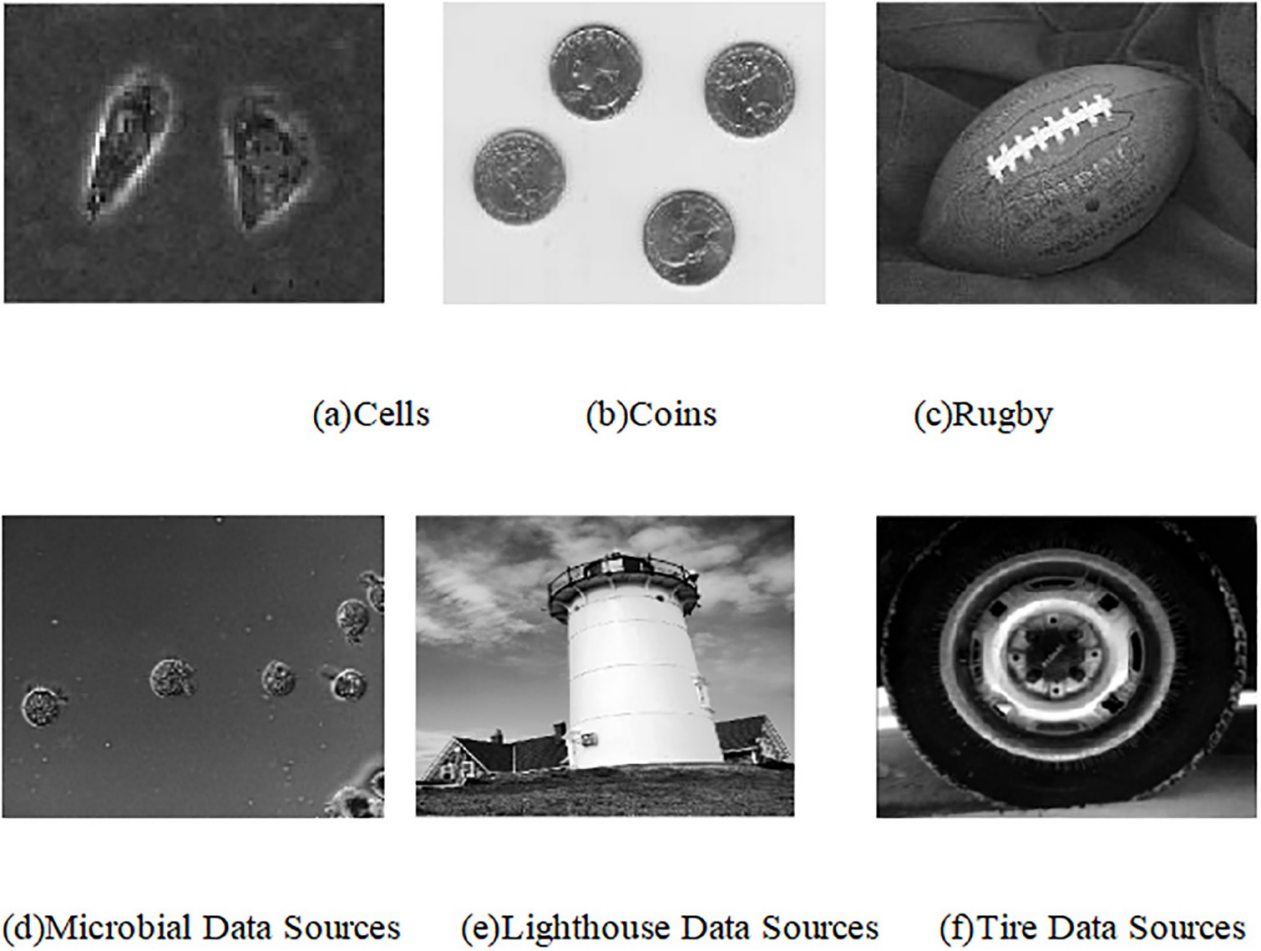
Experimental image. (a) Cells. (b) Coins. (c) Rugby. (d) Microbial Data Sources. (e) Lighthouse Data Sources. (f) Tire Data Sources.

In order to validate the effectiveness of the improved model without re-initialization level set, the results of image segmentation of the traditional model without re-initialization level set and the improved model without re-initialization level set proposed in this paper are discussed by using several sets of original target image data sources. The superiority and shortcomings of the proposed improved model image segmentation method are analyzed in depth through the extraction effect, time consumption and peak signal-to-noise ratio of the object edge contours of the original target image in the above two model segmentation results. The original standard target image used for the experimental simulation is shown in Fig 1 (The experimental images in Fig 1 are public picture data from Matlab software itself, which usually are located in C:\Program Files\MATLAB\R2018b\toolbox\images\imdata).

### 4.2 Analysis of simulation results

In order to make a more objective analysis of the experimental results of the model simulation without re-initialization level set before and after the improvement, the original target image object edge contour extraction results during the experimental simulation are compared by using the number of iterations n of 50,100,150,200 and 250 before and after the improvement. In general, the default first row is the experimental simulation image of the model before improvement, and the second row is the experimental simulation image of the model after improvement. In order to quantify and evaluate the improved no-weight initialization level set model more objectively, this paper quantifies the effectiveness of the improved no-weight initialization level set model in three dimensions: the time duration of the smoothing and noise reduction stage of the original target image, the edge contour extraction stage, and the signal-to-noise ratio of the object edge contour extraction map of the original target image obtained from the improved before and after model when the number of iterations reaches 250. The validity of the improved non re-initialization level set model was verified.

#### (1) Cellular data source simulation

**Fig 2:**
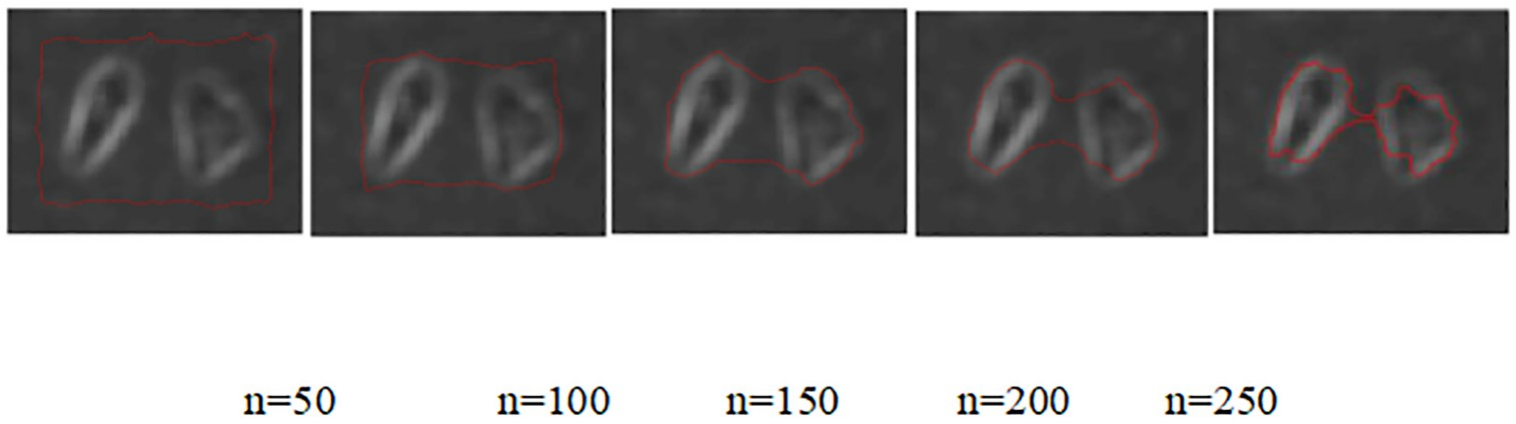
Comparison of cell edge extraction results by the model before and after improvement.

A comparison of the simulation experiments of the without re-initialization level set model before and after the improvement of the cellular data source images is shown in Fig 2:

According to the analysis of the cell data source edge contour extraction process by the improved before and after no re-initialization level set model shown in Fig 2, it can be seen that: when the number of iterations n = 50, there is no significant difference in the cell edge contour extraction effect between the improved before and after model; when the number of iterations n increases to 100, the improved before model has a certain degree of extraction of the four corners of the cell edge contour, and the improved after model has a somewhat inferior extraction effect on the lower left corner of the cell object edge contour; when the number of iterations n reached 150, the former model had a relatively complete extraction of the cell edge contour as a whole, and the improved model had a closer extraction of the cell edge contour as a whole, especially the left part; when the number of iterations a reached 200, the former model had a more complete extraction of the left side of the cell edge contour, but the post-model produced some over-segmentation of the cell edge contour; when the number of iterations n reached 250, the pre-model increased the over-segmentation range of the two cell edge contours without segmenting the edge contour of the two cell objects, and the post-model extracted a more complete edge contour of both cells.

**Fig 3:**
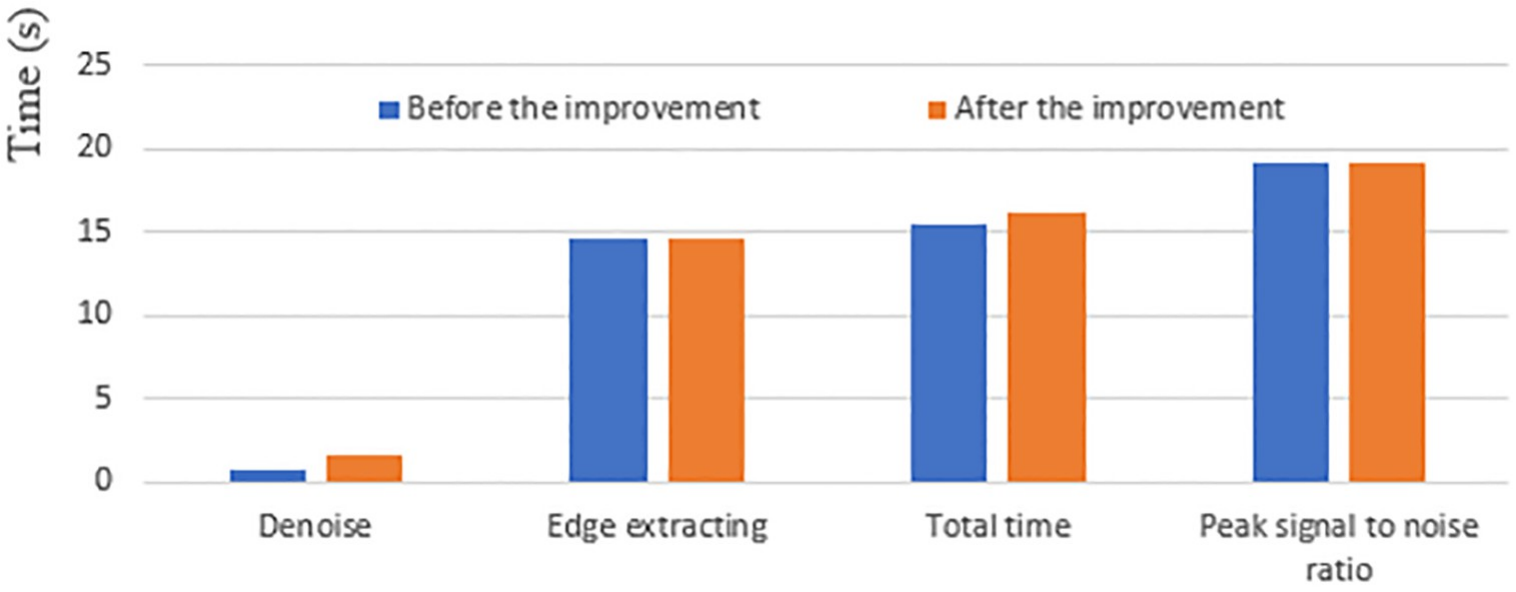
Graphical visual representation of Table 1.

The Table 1 provides more convincing data support for the further analysis of the improved model in this paper by counting the objective quantitative indexes of the no re-initialized model before and after the improvement. By calculating the peak signal-to-noise ratio for the selected regions {[5:145], [145:160]} of the cell edge contour extraction maps before and after the improvement, it can be seen that the peak signal-to-noise ratio of the improved model is higher than that of the pre-improved model by 0.031, thus verifying that the original target noise reduction effect of the improved model is better than that of the pre-improved model. The improved model only has good noise reduction effect on Gaussian noise in the noise reduction stage, so the noise reduction time of the improved model is lower than that of the improved model by 0.8709s. The time of the edge extraction stage of the improved model is less than that of the improved model by 0.102s, and the total time is higher than that of the improved model by 0.7689s. Combined with the overall analysis of Fig 2 and Table 1, the improved model has good extraction of cell edge contours, and good noise reduction for the original cell data source images; further optimization and improvement are needed in the noise reduction time phase. Graphical visual representation of Table 1 is showed in Fig 3.

**Table 1:** Quantitative metrics for weightless initialization models before and after cellular data source improvement.

Model	Noise reduction	Edge extraction	Total length	Peak Signal to Noise Ratio
	Duration in seconds (s)			[5:145],[145:160]
Before improvement	0.729	14.724	15.453	19.1863
After improvement	1.5999	14.622	16.2219	19.2173

#### (2) Coin data source simulation

**Fig 4:**
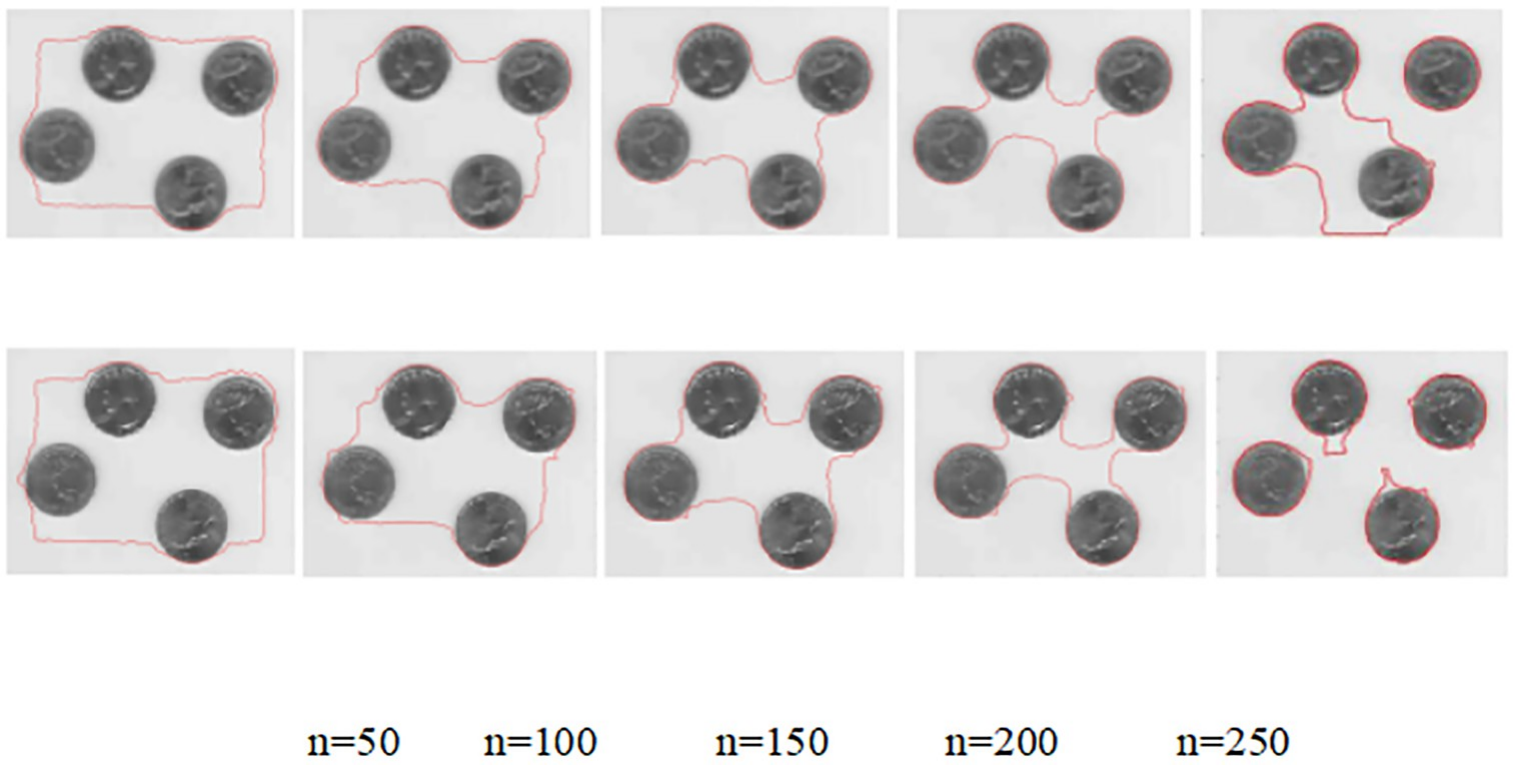
Comparison of edge extraction results of coin data source images before and after the improved model.

A comparison of the original target image improvement method for coin data sources with the traditional no re-initialization level set model simulation experiments is shown in Fig 4:

Fig 4 compare the coin edge contour maps before and after the improvement for the coin data source at the number of iterations n = 50, 100, 150, 200 and 250 respectively. A visual observation and analysis of the first, second, third and fourth groups of Fig 4 shows that there is not much difference in the coin edge contour extraction of the models before and after the improvement. When the number of the iterations is n = 250, the pre-model only extracts one coin edge contour in the upper right corner completely, and the other three coin edge contours are not segmented; while the post-model has basically segmented the four coin edge contours completely and obtained the contour extraction results that are closer to the real edges of the four coins.

**Fig 5:**
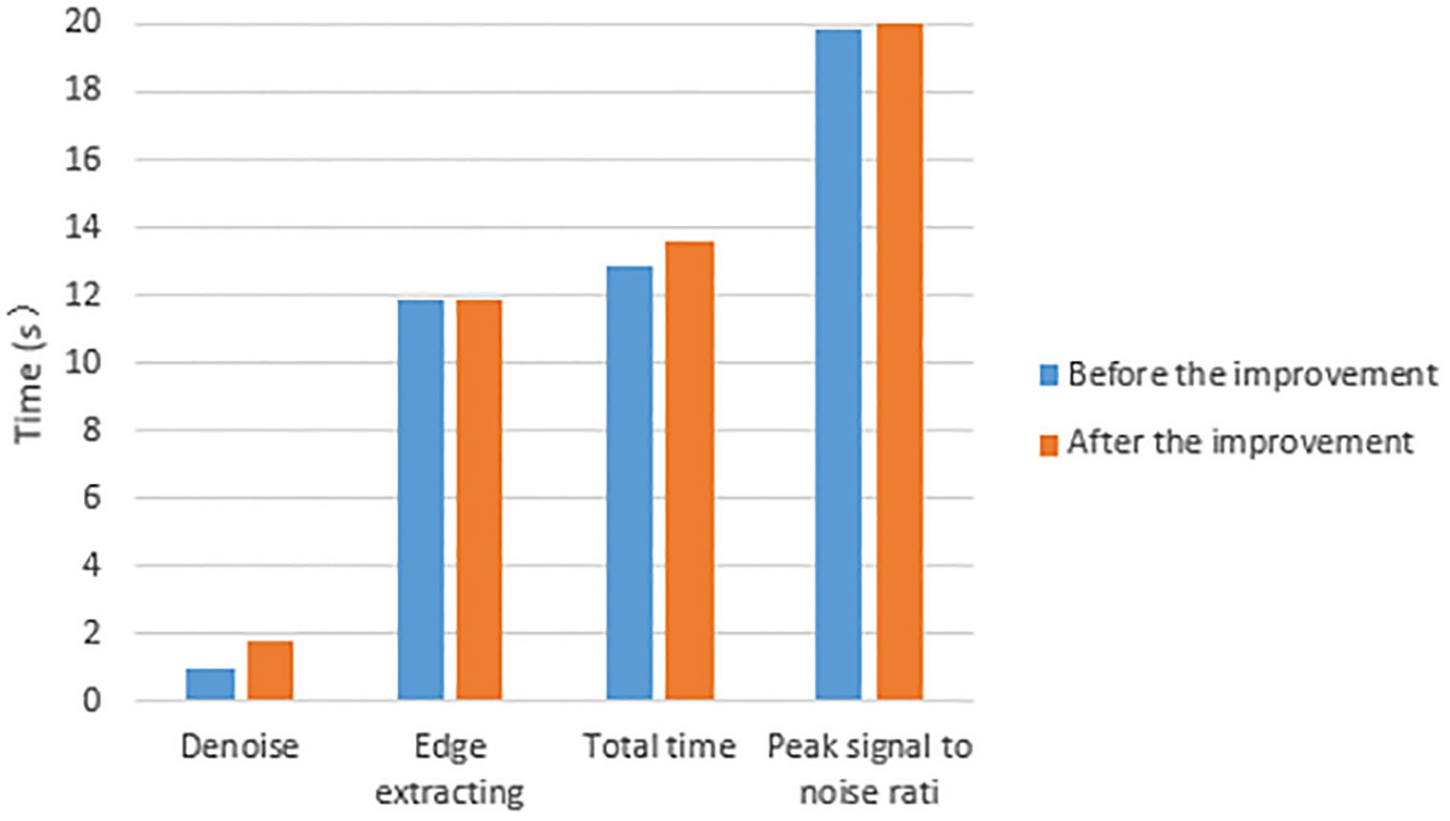
Graphical visual representation of Table 2.

The Table 2 provides statistics on the time consumed by the coin data source for noise reduction, edge extraction and the total duration of the model before and after the improvement. The improved model also has a certain degree of effect on the noise other than Gaussian noise in the original target image, so the time duration of the improved model in the noise reduction stage is higher than that of the pre-improvement model by 0.8334s; the time duration of the edge extraction stage is lower than that of the pre-improvement model by 0.061s. Overall, the time duration of the edge contour extraction of the coin source by the improved model is higher than that of the pre-improvement model by 0.7724s. Using the original coin source data map as the reference map, the regions of {[20:100],[40:100]} were selected, and the peak signal-to-noise ratio was calculated by selecting regions of equal size for the coin edge contour extraction maps of the improved before and after model, and the improved model is higher than the improved model by 0.6012, thus the image noise reduction results of the improved model are verified. Graphical visual representation of Table 2 is showed in Fig 5.

**Table 2:** Quantitative metrics for no re-initialization models before and after improvement to the coin data source.

Model	Noise reduction	Edge extraction	Total length	Peak Signal to Noise Ratio
	Duration in seconds (s)			[20:100],[40:100]
Before improvement	0.925	11.909	12.834	19.8235
After improvement	1.7584	11.848	13.6064	20.4247

#### (3) Rugby data source simulation

**Fig 6:**
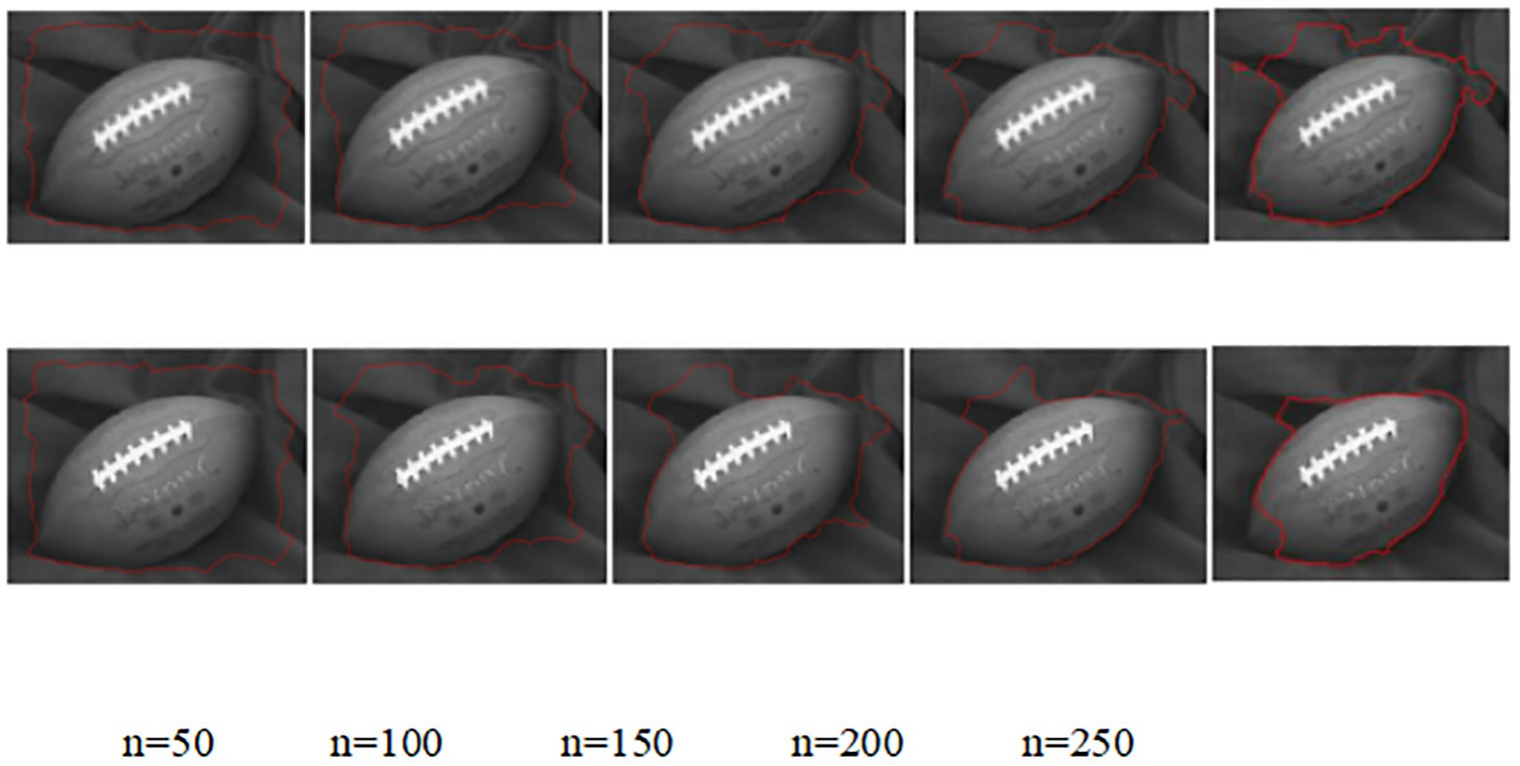
Comparison of edge extraction results rugby data source images by the improved before and after model.

A comparison of the experimental results of the simulation of the no re-initialization level set model before and after the improvement of the original target images of the rugby ball data source is shown in Fig 6:

Rugby ball data source at the number of iterations n = 50 and 100 in Fig 6, respectively, the model before and after the improvement is slightly different in the broad object edge contour range extraction. When the number of iterations n = 150, the improved model in the third set of simulated images is significantly less effective than the improved model in extracting contours to the left of the top of the rugby ball. When the number of iterations n = 200, the improved model achieved significantly better edge contour extraction results on the fourth set of simulated experimental images, especially when the position on the right slice of the rugby ball was basically close to the rugby ball edge contour of the original target image. When the number of iterations n = 250, the improved model showed over-segmentation on the left bottom of the rugby ball in the fifth group of experimental simulations, but still achieved good edge extraction results overall; the improved model successfully avoided the over-segmentation phenomenon of the improved model, and the edge contour extraction on the left side of the rugby ball deviated seriously from the real edge of the rugby ball in the original target image.

**Fig 7:**
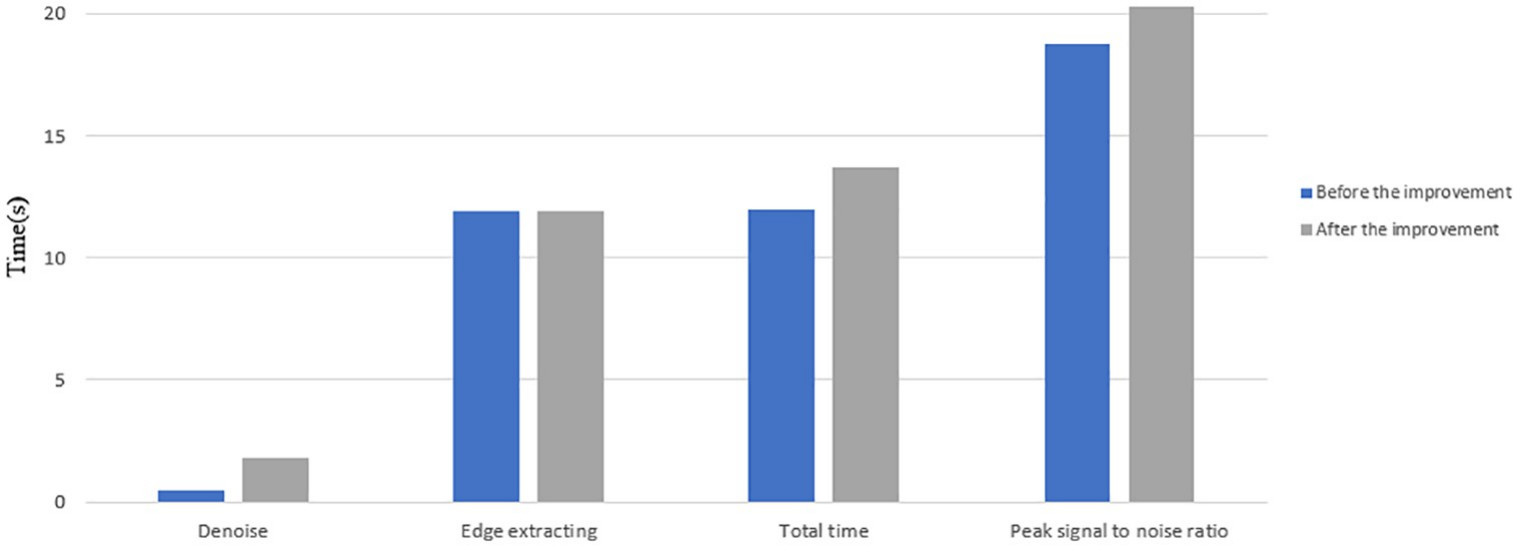
Graphical visual representation of Table 3.

The objective quantitative indicators of the rugby ball data source in the before and after improvement model without re-initialization are counted and presented in the Table 3. The original target image of the rugby ball was used as the reference image for the rugby ball edge result extraction maps of the before and after improvement model, and the peak signal-to-noise ratio was calculated for the reference, before and after improvement model edge contour extraction maps according to the principle of equal scale selection. According to the comparison of the peak ratios of image regions with selected regions {[20:80],[20:60]} provided in the Table 3, it is found that the peak signal-to-noise ratio of the edge contour extraction map of the improved model is higher than that of the improved model by 1.3368s, which validates the effectiveness of the improved model in the smoothing and noise reduction stage. The comparison of the noise reduction, edge extraction and total time of the improved model showed that the time of the target image processed by the improved model in the noise reduction stage was slightly lower than that of the improved model by 0.016s; the time of the noise reduction stage was higher than that of the improved model by 1.698s, and the total time was higher than that of the improved model by 1.3208s. The time consumed by the improved model in the smoothing and noise reduction phase of the original target image needs to be further optimized. Graphical visual representation of Table 3 is showed in Fig 7.

**Table 3:** Quantitative metrics of the without re-initialization model before and after the improvement of the rugby data source.

Model	Noise reduction	Edge extraction	Total length	Peak Signal to Noise Ratio
	Duration in seconds (s)			[20:80],[20:60]
Before improvement	0.4515	11.901	12.3525	18.7508
After improvement	1.7883	11.885	13.6733	20.2691

#### (4) Simulation of microbial data sources

**Fig 8:**
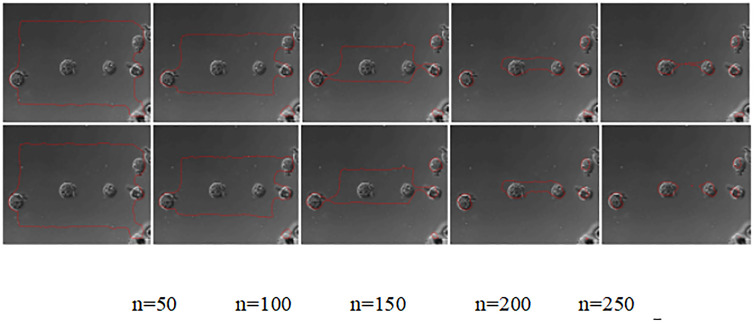
Comparison of microbial edge extraction results from the model before and after improvement.

A comparison of experimental simulations of the non-re-initialized level set model before and after improvement of the microbial data source images is shown in Fig 8:

Fig 8 shows the stage by stage process of object edge contour extraction from microbial data source, comparing the extraction results of before and after the improved model, which can verify the effectiveness of the improved model object edge contour extraction more intuitively. According to Fig 8, the difference between the before and after model is very small when the number of iterations n = 50, 100 and 150; when the number of iterations n = 200, the improved model has a further extraction effect for the middle two objects compared with the before model; when the number of iterations n = 220, the improved model has a relatively complete extraction result for each object edge contour. For n = 220 iterations, the improved model has a more complete extraction result for each object’s edge contour, while the former model has not yet segmented the middle two objects’ edge contours.

**Fig 9:**
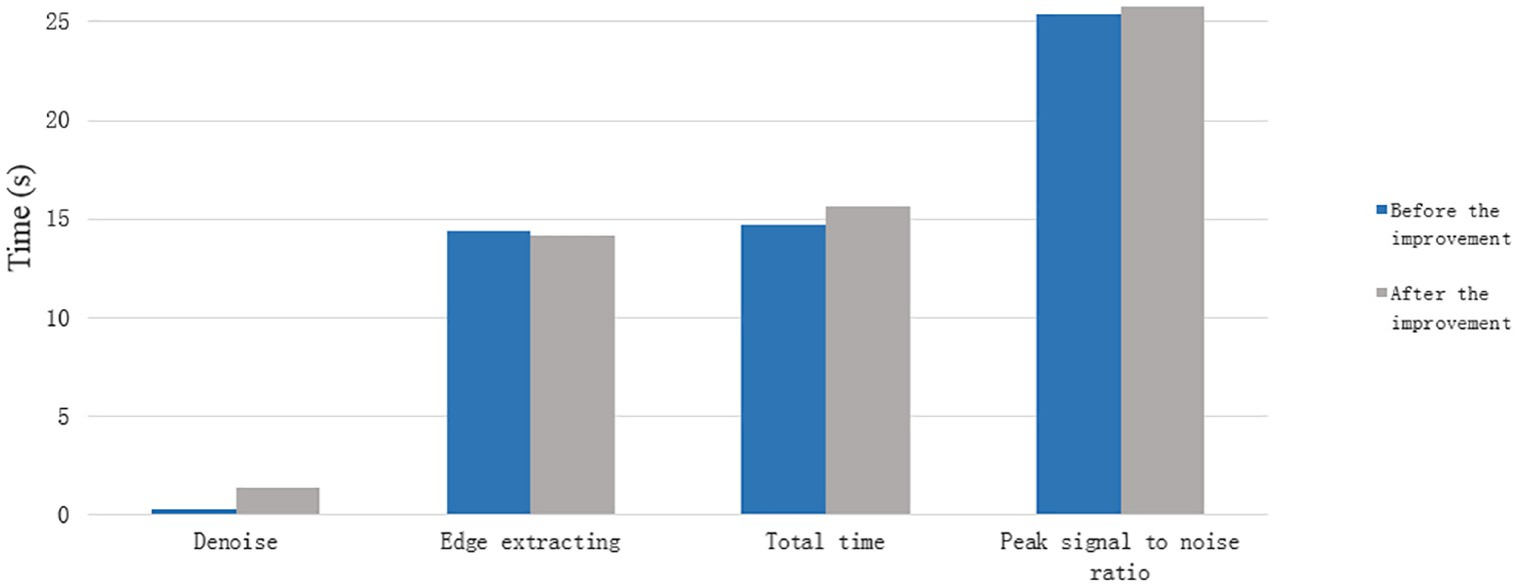
Graphical visual representation of Table 4.

The Table 4 provides convincing data support for the improved model in this paper by analyzing the four dimensions of noise reduction, edge extraction, total time and peak signal-to-noise ratio of selected regions before and after the improved model. The improved model takes 1.1170s longer than the pre-improvement model in the noise reduction stage because it takes into account the spatial and range domains of the original target image pixels, the edge extraction time is 0.242s less than the pre-improvement model; the total time is still 0.875s longer than the pre-improvement model. From the selected regions {[1:105],[1:115]} for peak signal-to-noise ratio comparison, the peak signal-to-noise ratio of the improved model was higher than that of the pre-model by 0.3943. Combined with Fig 8 and Table 4, the improved model was more effective in extracting microbial edge contours and had good noise reduction effect on the original microbial data source images. Graphical visual representation of Table 4 is showed in Fig 9.

**Table 4:** Quantitative metrics for non-reinitialized models before and after microbial data source improvement.

Model	Noise reduction	Edge extraction	Total length	Peak Signal to Noise Ratio
	Duration in seconds (s)			[1:105],[1:115]
Before improvement	0.3105	14.428	14.7385	25.4111
After improvement	1.4275	14.186	15.6135	25.8054

#### (5) Lighthouse data source simulation

**Fig 10:**
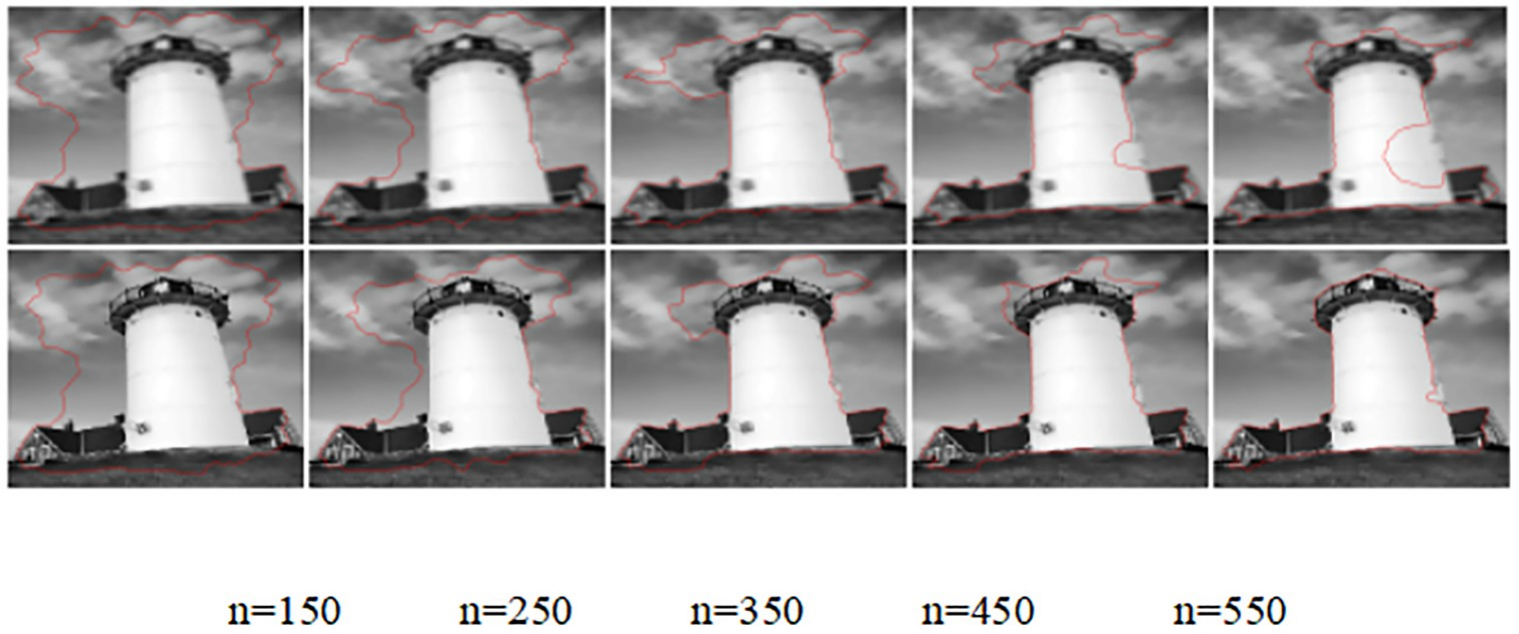
Comparison of the edge extraction results of the lighthouse data source images before and after the improved model.

A comparison of the original target image improvement method for the lighthouse data source with the traditional no re-initialization level set model simulation experiments is shown in Fig 10:

Fig 10 shows the extraction results of the outer edge of the lighthouse and the surrounding houses for n = 150,250,350,450 and 550 iterations of the lighthouse data source. By comparing the first three sets of Fig 10, it can be seen that the before and after model slightly inferior to the improved model in terms of the extraction results of the object edges. When the number of iterations n = 450, the right lower part of the lighthouse in the improved model has obvious distortion; When the number of iterations n = 550, the improved model has extracted the outer edge contour of the lighthouse and the surrounding houses more completely, and the edge contour extraction result of the improved model deviates from the real edge of the object seriously.

**Fig 11:**
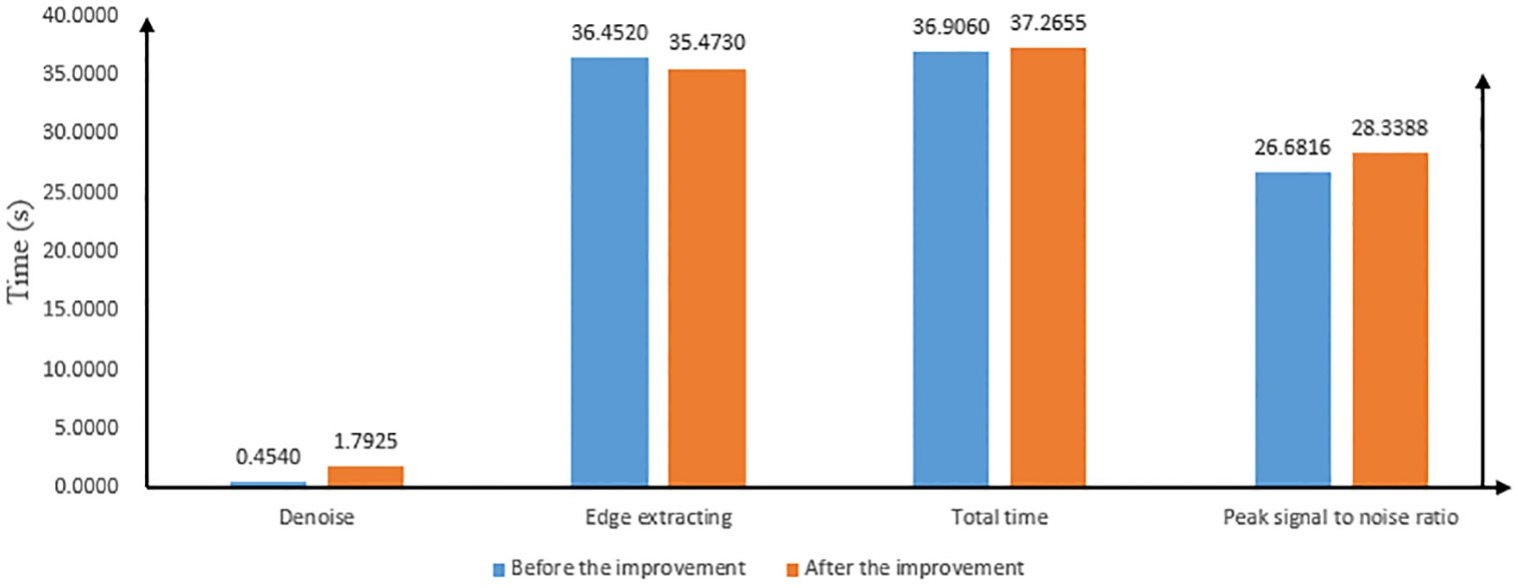
Graphical visual representation of Table 5.

The Table 5 provides statistics on the time consumed by the model before and after the improvement in noise reduction, edge extraction and total duration of the lighthouse data source. The improved model also has a certain degree of smooth noise reduction effect on other noises except Gaussian noise, so the time duration of the noise reduction stage of the improved model is higher than that of the pre-improved model by 1.3385s. The time duration of the edge extraction stage is lower than that of the pre-improved model by 0.979s. Overall, the time duration of the edge contour extraction of the lighthouse data source image of the improved model is higher than that of the pre-improved model by 0.3595s. With the original lighthouse source data image as the reference, the {[1:165],[150:185]} area is selected, and the peak signal-to-noise ratio is calculated for the same size area selected for the lighthouse and the outer edge contour extraction map of the surrounding houses of the model before and after the improvement, and the improved model is higher than the model before the improvement by 1.6572. Graphical visual representation of Table 5 is showed in Fig 11.

**Table 5:** Quantitative metrics of the no-weight initialization model before and after the improvement of the lighthouse data source.

Model	Noise reduction	Edge extraction	Total length	Peak Signal to Noise Ratio
	Duration in seconds (s)			[1:165],[150:185]
Before improvement	0.4540	36.452	36.906	26.6816
After improvement	1.7925	35.473	37.2655	28.3388

#### (6) Tire data source simulation

**Fig 12:**
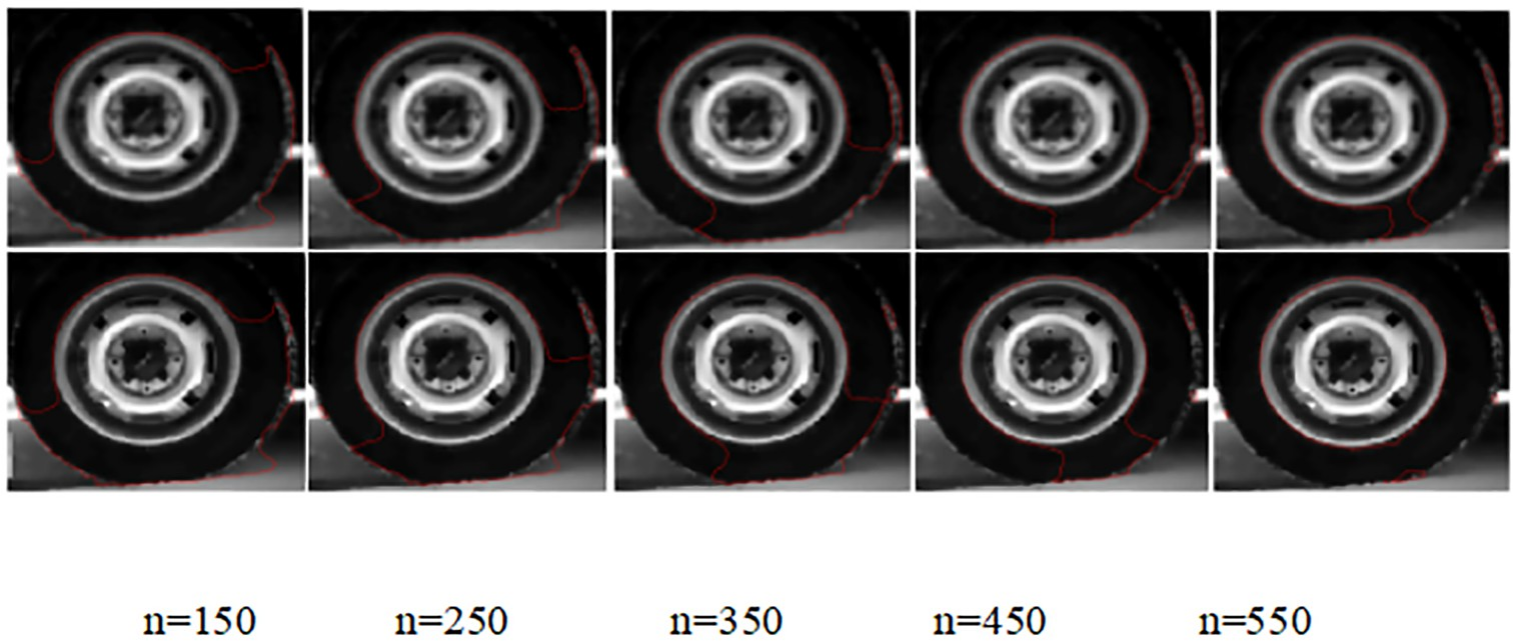
Comparison of the edge extraction results of the lighthouse data source images by the model before and after improvement.

A comparison of the experimental results of the simulation of the no re-initialization level set model before and after the improvement of the original target images of the type data source is shown in Fig 12:

Fig 12 compares the tire inner edge contour extraction results before and after the improvement for the tire data source at the number of iterations n = 150, 250, 350, 450 and 550. The visual analysis and observation of the first four groups in Fig 12 show that there is no significant difference in the inner edge profile extraction of tires between the before and after model. When the number of iterations is n = 550, the inner lower right corner edge of the tire is not completely extracted by the improved model; the improved model obtains the real edge extraction results closer to the inner side of the tire.

**Fig 13:**
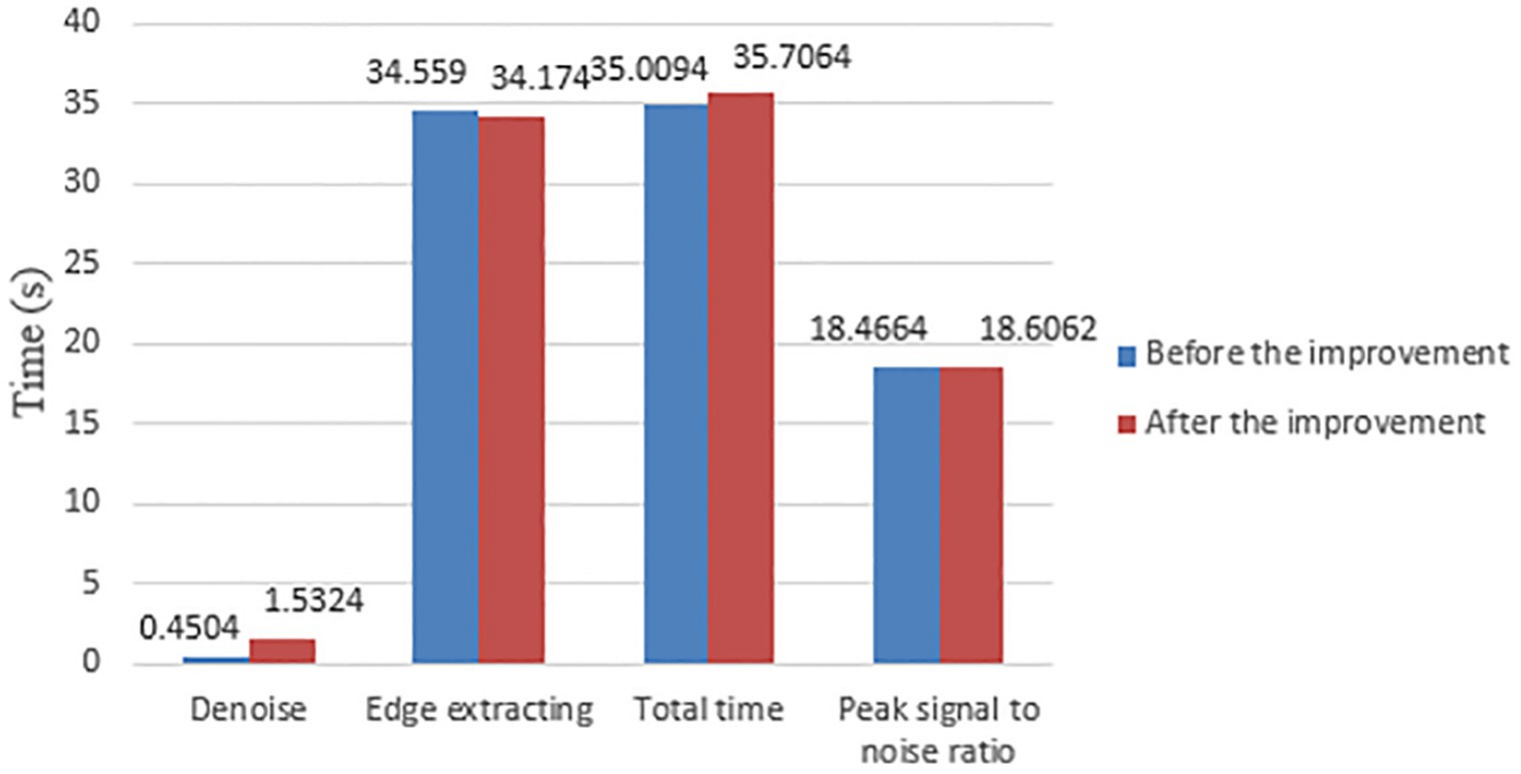
Graphical visual representation of Table 6.

Table 6 quantifies the edge contour extraction results of the improved before and after model in four dimensions: noise reduction, edge extraction, total time, and peak signal-to-noise ratio of the selected region. In the three dimensions of noise reduction, total time, and peak signal-to-noise ratio of selected region, the improved model is higher than the pre-model by 1.082s, 0.697s, and 0.1398, respectively; the time spent in the edge extraction stage is higher than that of the post-model by 0.385s. Combined with Fig 12 and Table 6, the effectiveness of the algorithm of the improved model is verified. Graphical visual representation of Table 6 is showed in Fig 13.

**Table 6:** Quantitative indicators of the weightless initialization model before and after improvement of the tire data source.

Model	Noise reduction	Edge extraction	Total length	Peak Signal to Noise Ratio
	Duration in seconds (s)			[1:115],[1:125]
Before improvement	0.4504	34.559	35.0094	18.4664
After improvement	1.5324	34.174	35.7064	18.6062

The experimental simulation results show that the improved weightless initialized level set model algorithm proposed in this paper effectively improves the ambiguity of the traditional weightless initialized level set model for the original target image object edges and the overall noise reduction, and from the perspective that the time spent on the extraction efficiency of the original target image object edge contours is less than that of the traditional weightless initialized level set model before the improvement, the accuracy and effectiveness of the improved weightless initialized level set model algorithm proposed in this paper are confirmed.

## 5. Conclusion

Aiming at the current situation that the traditional weightless initialized level set model is not ideal for extracting edge contours in a given original target image object, this paper proposes an improved algorithm of weightless initialized level set model based on bilateral filters, taking the classical weightless initialized level set as the entry point of the study. The improved weightless initialized level set is obtained by replacing the Gaussian filter in the image smoothing and noise reduction stage. The improved weightless initialized level set model effectively considers the pixel values between the contour points of the object edges of the original target image in the noise reduction processing stage of the original target image, so that the information of the spatial proximity weight domain and the pixel gray value weight domain values of the current evolving points are considered comprehensively. In this paper, the original target image object edge contours are effectively extracted by the introduction of bilateral filters in combination with the traditional weightless initialized level set model algorithm proposed by Li model et al. In addition, a comparison of the runtime of the traditional unweighted initialized level set model and the improved unweighted initialized level set model is carried out in stages of experimental simulation. Comparing the experimental results of image data sources of the improved model before and after the improvement, we can see that the improved model obtains clearer target images after smoothing and noise reduction of the original target images; at the same time, the improved model extracts the selected object edge contours more closely to the real edges of the objects.

Through the objective quantitative evaluation of the object edge contour target images before and after the improvement, it is easy to find that the improved model still needs further optimization and improvement. The time duration of the smoothing and noise reduction stage of the no-weight initialization level set model before and after the improvement is higher than that of the no-weight initialization level set model before the improvement, and it is necessary to further explore how to improve the smoothing and noise reduction time of the improved model without affecting the smoothing and noise reduction effect of the original target image in the subsequent research.
